# Estimation of model accuracy by a unique set of features and tree-based regressor

**DOI:** 10.1038/s41598-022-17097-z

**Published:** 2022-08-18

**Authors:** Mor Bitton, Chen Keasar

**Affiliations:** Department of Computer Science, Ben Gurion University, Be’er Sheva, Israel

**Keywords:** Computational biology and bioinformatics, Machine learning, Protein structure predictions

## Abstract

Computationally generated models of protein structures bridge the gap between the practically negligible price tag of sequencing and the high cost of experimental structure determination. By providing a low-cost (and often free) partial alternative to experimentally determined structures, these models help biologists design and interpret their experiments. Obviously, the more accurate the models the more useful they are. However, methods for protein structure prediction generate many structural models of various qualities, necessitating means for the estimation of their accuracy. In this work we present MESHI_consensus, a new method for the estimation of model accuracy. The method uses a tree-based regressor and a set of structural, target-based, and consensus-based features. The new method achieved high performance in the EMA (Estimation of Model Accuracy) track of the recent CASP14 community-wide experiment (https://predictioncenter.org/casp14/index.cgi). The tertiary structure prediction track of that experiment revealed an unprecedented leap in prediction performance by a single prediction group/method, namely AlphaFold2. This achievement would inevitably have a profound impact on the field of protein structure prediction, including the accuracy estimation sub-task. We conclude this manuscript with some speculations regarding the future role of accuracy estimation in a new era of accurate protein structure prediction.

## Introduction

Protein structure prediction (*PSP*) has been a major challenge in computational biology for half a century already^1,2^. Given a target protein sequence (hereafter referred to as ”*target*”), PSP methods aim to provide a three-dimensional model of the protein molecule. Such models help biologists build their theories and design their experiments. They provide a partial remedy to the high cost and much labor required for the experimental determination of structures. Typically, prediction methods generate many alternative structural models for each target. These models have diverse qualities even if generated by the same method, and often the best structural models of different targets are generated by different prediction methods. Unfortunately, large sets of alternative models do not provide much insight into biological problems, and the identification of the best models has been recognized early on^3^ as an essential PSP sub-task, known as Estimation of Model Accuracy (*EMA*, aka *QA*). EMA methods come in two flavors: local, assigning an accuracy measure to each residue of a model^4–7^, and global, assessing the qualities of complete models^8,9^. Often the former, in addition to its own merit, serves as a stepping stone to the latter^10–12^.

This manuscript presents MESHI_consensus, a new EMA method, with state-of-the-art performance. Specifically, MESHI_consensus aims to predict the similarity of protein models to the corresponding native structures in terms of the zero to one Global Distance Test Total Score (*GDT_TS*), which assigns a score of one to models that are very similar to the native structure and lower scores to models that are less similar. A score close to zero indicates an irrelevant model.

In the last two decades, the Critical Assessment of Protein Structure Prediction (CASP), a biannual and community-wide series of prediction experiments^13–15^ monitor the performance of prediction methods and accelerate their development. In each experiment, CASP organizers collect around a hundred targets at the final stages of their structural determination, and challenge researchers to submit blind predictions of the yet unknown structures. The assessment of these models, when the structures are finally determined, allows a reliable evaluation of prediction methods. Over the years CASP has become the de facto ”Gold Standard” of the PSP field, and the recent unprecedented performance of AlphaFold2 in 14th CASP experiment^16,17^ is commonly recognized as marking a new era in structural biology. The CASP experiments have several tracks for PSP sub-tasks, and since 2008, EMA is considered a CASP category^8,18–21^. The relevance of EMA in an era of high accuracy structural modeling is considered in the Discussion section below. CASP experiments play a two-fold role in the current study: our benchmark^22^ is based on structural models submitted to the 9th to 13th CASP rounds, and the 14th experiment is used to evaluate the new EMA method.

Since the early days of EMA^23,24^ and up until recently, the best performing methods have used the consensus (aka multi-model) approach, which considers structural similarity between independently generated models as an indication that they are likely to be all similar to the unknown native structure^8,18,19,25^. Not withstanding their power however, consensus EMA does not provide any insight about the actual physics of protein folding or the essence of being a correct structure. Further, it cannot be applied to a single model, and fails to identify exceptionally good models. These limitations motivate an alternative, single-model, approach that considers the internal properties of a single model structure (e.g., estimated energy and compactness) as well as its compatibility with one dimensional predictions of secondary structure and solvent accessibility, which are based of multiple sequence alignments (MSAs)^26–29^. Recently, compatibility with contact predictions derived from deep multiple sequence alignments seems to be a game changer, considerably improving EMA performance and allowing single-model methods to outperform consensus based ones^21,30,31^.

Most recent EMA methods use machine learning (*ML*) algorithms, including neural networks^32–34^, SVM^35–37^, and tree-based methods^38^, to create a statistical model that combines measurable features into a single number, which estimates the quality of a structural model^35,39^. To this end, ML algorithms use datasets of annotated structural models and learn the intricate relations between the features and model quality. Specifically, the EMA methods use model structures to produce meaningful features, such as statistical pairwise potentials^40,41^ and consensus-derived terms^42^. These features constitute the input for regression models^9,43^ that integrate them into a single score. An emerging Deep learning-based approach eliminates the distinction between feature generation and and learning of statistical model. It uses convolutional^6,44,45^ and graph^46,47^ neural networks to derive the scores directly from elementary features such as atom/residue types and distances. Higher order features, analogous to energy terms and other traditional features, emerge as information flows throw the the networks layers.

Our method, MESHI_consensus, uses a tree-based machine-learning algorithm to estimate model qualities from 982 structural and consensus features.

## Methods

The following sections introduce the basic components of MESHI_consensus. We first present our benchmark, a dataset of targets and structural models thereof from previous CASP experiments, and the features derived from them. Then we describe the performance measures that guided the development of the ML model, as well as the model design procedure, which includes regressor and hyper-parameters selection. Finally, we present a data filtering process that reduces training set noise.

### Structural models dataset

We trained and evaluated our method using a dataset that consists of 73,053 single-chain models, which were generated as blind server predictions of 345 CASP9-CASP13 targets (2010–2018) (Table S1) an extension of the dataset used in^22^. These targets are a non-redundant subset of the $\approx$430 targets of these CASP experiments. To this end, targets were considered redundant, and discarded, if a newer target was strictly similar by either sequence ($E-value < 10^{-3}$) or structure (more than a half of the residues could be structurally aligned by the iterative magic fit method of Swiss-PDB-Viewer^48,49^). Duplicated models, having identical conformations as other ones (typically from different servers of the same group), were identified and removed.

Many server-generated models include clashes (too short distances) between atoms and other structural distortions, such as deviations from correct bond lengths and angles. Thus, before feature extraction, each structural model was subjected to energy minimization using the MESHI molecular modeling package^50^. The energy function includes strong spatial constraints, and most distortions are removed with negligible structural changes.

The test set of this study includes 67 CASP14 targets (10,889 structural models), which were predicted by modeling servers during the CASP14 experiment (May–August, 2020). Both model generation and the estimation of their accuracy by MESHI_consensus web server were done in a blind fashion before their structures became available. After the models were downloaded from the CASP14 website they were energy minimized by MESHI package and their features were fed to the MESHI_consensus model.

#### Features dataset

In this study, each structural model is represented by a vector of 982 features. These features may be divided into two broad classes: structural model-features, and target-features that modulate the former.

#### Structural model-features 

The following features are calculated using the MESHI package^50^.

- Basic features: 142 features derived solely from single model structures^51^. They include a mix of commonly used and novel knowledge-based energy terms (some of which are unpublished yet). These terms include pair-wise atomic potentials^40,52,53^, torsion angles^54^, hydrogen bonds, and hydrogen bond patterns^55^, solvation terms, ”meta” energy terms that consider the distribution of other terms within the protein atoms, an extended radius of gyration that takes into account different classes of amino acids (polar vs. non-polar, secondary structure elements vs. coil region, etc.), and compatibility of the models with solvent exposure prediction^56^, and with 3-, 8- and 13-classes secondary structure predictions^57–59^.
- Consensus features: seven features (Eqs. 1–7) that represent the similarities between a model of a specific target and the other models of the same target. These terms are calculated as follows: $\forall d \varvec{\in } T$, where *T* is the set of structural models of some target.
  1 $$\begin{aligned} g d t_{i}\_\text{consensus }(d)=\frac{1}{|T|} \sum _{s \in T} g d t_{i}(d, s) \\ \text{ where } \text{ i } \in \{{\mathbf {1}}, {\mathbf {2}}, {\mathbf {4}}, {\mathbf {8}}\} \end{aligned}$$
  2 $$\begin{aligned} g d t_{-} t s_{-} \text{consensus }(d)=\frac{1}{|T|} \sum _{s \in T} g d t_{-} t s(d, s) \end{aligned}$$
  3 $$\begin{aligned} g d t_{-} h a_{-} \text{consensus }(d)=\frac{1}{|T|} \sum _{s \in T} g d t_{-} h a(d, s) \end{aligned}$$
  4 $$\begin{aligned} r m s_{-} \text{consensus }(d)=\frac{1}{|T|} \sum _{s \in T} R M S D(d, s) \end{aligned}$$
  Where: $$\forall d, s \varvec{\in } T$$
  5 $$\begin{aligned} g d t_{j \in \{0.5,1,2,4,8]}\left( d, s\right) = \text{ The}\, \text{maximal} \,\text{fraction}\, \text{of} \, {\varvec{s}}\, \text{residues}\, \text {that} \, \text{are} \, \text {less} \, \text{than}  \,j\, {{^\circ\!\!\,\,\!\!\!{\rm A}} }\, \text {form} \,\text {the} \, \text {corresponding} \, \text {residues} \, \text {of} \, {\varvec{d}} \,\text{after} \, \text {superposition}. \end{aligned}$$
  6 $$\begin{aligned} g d t_{-} t s(d, s)=\frac{\sum _{i \in \{1,2,4,8\}} g d t_{i}(d, s)}{4} \end{aligned}$$
  7 $$\begin{aligned} g d t_{-} h a(d, s)=\frac{\sum _{k \in \{0.5,1,2,4\}} g d t_{k}(d, s)}{4}\\ \end{aligned}$$

#### Target-features

**Figure 1:**
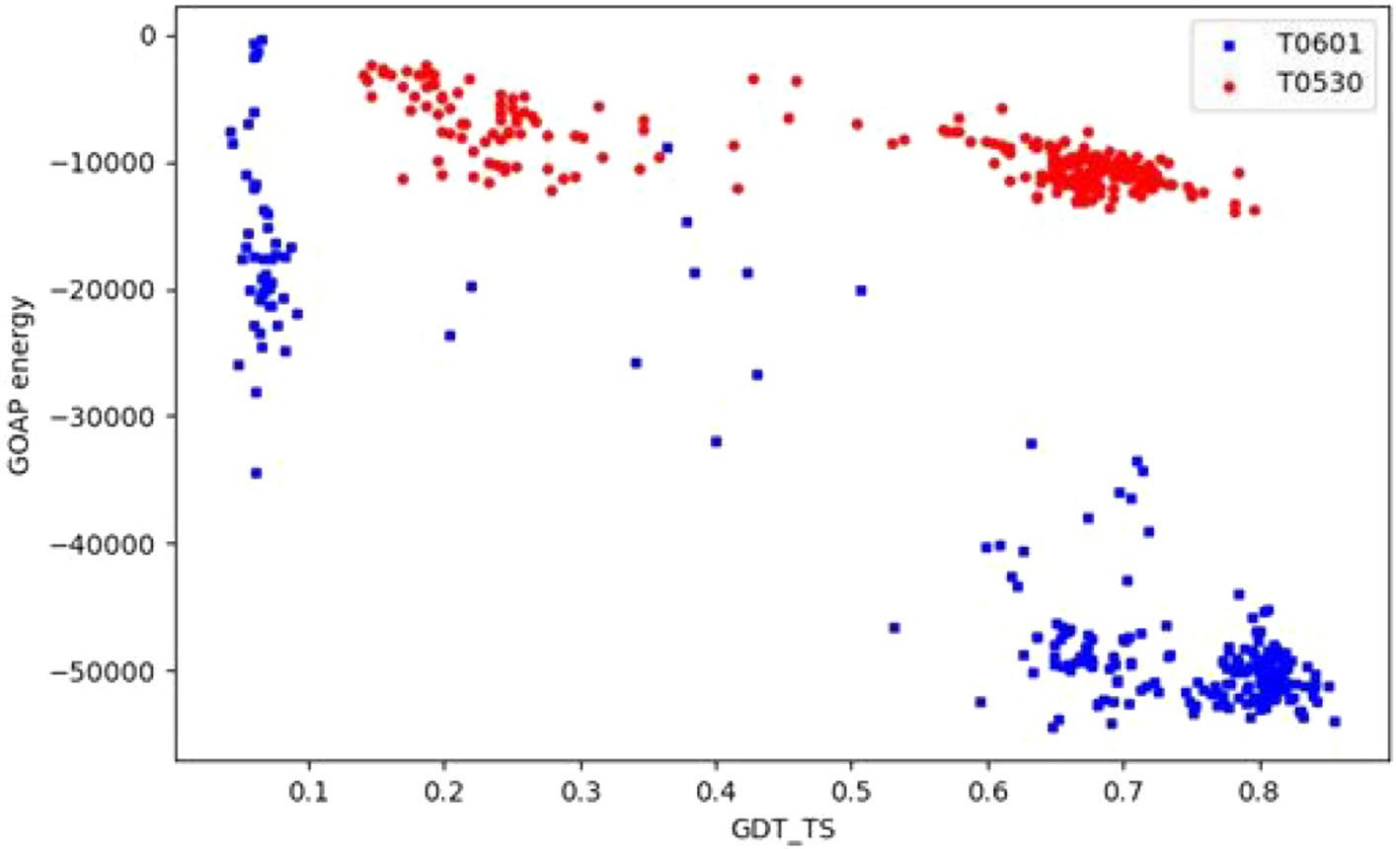
Target characteristics bias feature values. Each point in the figure represents the ”GOAP”S energy^60^ and accuracy of a single structural model. Models of the two CASP targets T0601 and T0530 are depicted by blue and red points respectively. Accuracy is measured in GDT_TS between the models and the native structures, that is $g d t_{-} t s(d, n)$, Eq. (6), where *n* is the native structure of that target). ”GOAP” energy term is a strong feature and it (anti) correlates well with the accuracies of both targets. Yet, given a feature value range (e.g., around–10,000), the qualities of the two proteins are very different.

EMA datasets are organized in two levels; the objects that we study, and whose accuracies we predict are structural models. Yet each model belongs to a specific target (with no overlap between the targets). Each target is characterized by a unique sequence, which is shared by all its models, and a unique native structure. Thus, the mapping of features to model qualities may be biased by target characteristics such as length and chemical composition (amino acid sequence), which differ between targets but are typically identical in most models of a given target. Therefore, feature distributions and their relation to model quality differ between targets (Fig. 1). Further, some training set targets may be less informative than others, with respect to certain features, due to specific characteristics such as ligand binding.

We use this domain knowledge to generate target-specific features that allow the learning process to modulate the outcome of the model features:

- One-hot encoding of target name: binary features (one per target). That is, for each target *T* of the training set, there is a feature $OH_T$ such that $OH_T = 1$ for all the models of *T* and 0 otherwise. When positioned in a node of a decision tree, $OH_T$ splits the leaves of the sub-tree to *T* and $non-T$, rendering the features in the nodes of the *T* sub-tree practically meaningless. Interestingly the training process does make use of this ability to eliminate the effect of specific features in specific targets.
- Z-score, a normalized (zero mean and standard deviation of one) version of each basic feature, based on the target’s mean and standard deviation.
- Amino acid composition:
  - 20 features for the frequency of each amino acid in the sequence of the target.
  - 6 features for the frequency of amino acids with certain properties in the sequence: Positive charged, negative charged, aromatic, polar, and non-polar.

Combining all the feature vectors of the structural models dataset creates a features dataset.

### Performance measures

We aim to predict the accuracy of structural models in terms of GDT_TS between the models and the native structures, that is $g d t_{-} t s(d, n)$ (Eq. 6) where *n* is the native structure of that target. Specifically, we use three performance criteria:

1. Root mean square of prediction errors (*RMSE*)—the per-target distance between the prediction values and the observed (true) values.
2. LOSS - for each target, the difference between the quality of the best model (highest observed GDT_TS) and the quality of the top-ranking model.
3. 5-LOSS - for each target, the minimum difference between the quality of the best model (highest observed GDT_TS) and the qualities of the five top-ranking models.

For dataset models, method performance is estimated by the median of 345 Leave-One-Target-Out cross-validation experiments (one per dataset target). In each experiment, the statistical model is trained using all the targets except one, which serves as the test set. This strategy is computationally expensive but reduces biases, and in a sense simulates the real-world scenario, where we learn from all the models of targets whose native structures are known and assess the model qualities of a target whose structure is yet unknown.

### Method design

The design of these EMA methods aimed to optimize two performance criteria: the median of the per-target RMSE and median LOSS. We used Leave-One-Target-Out cross-validation experiments to choose the regressor, its hyper-parameters, and the data filtering strategy.

#### Regressor

**Figure 2:**
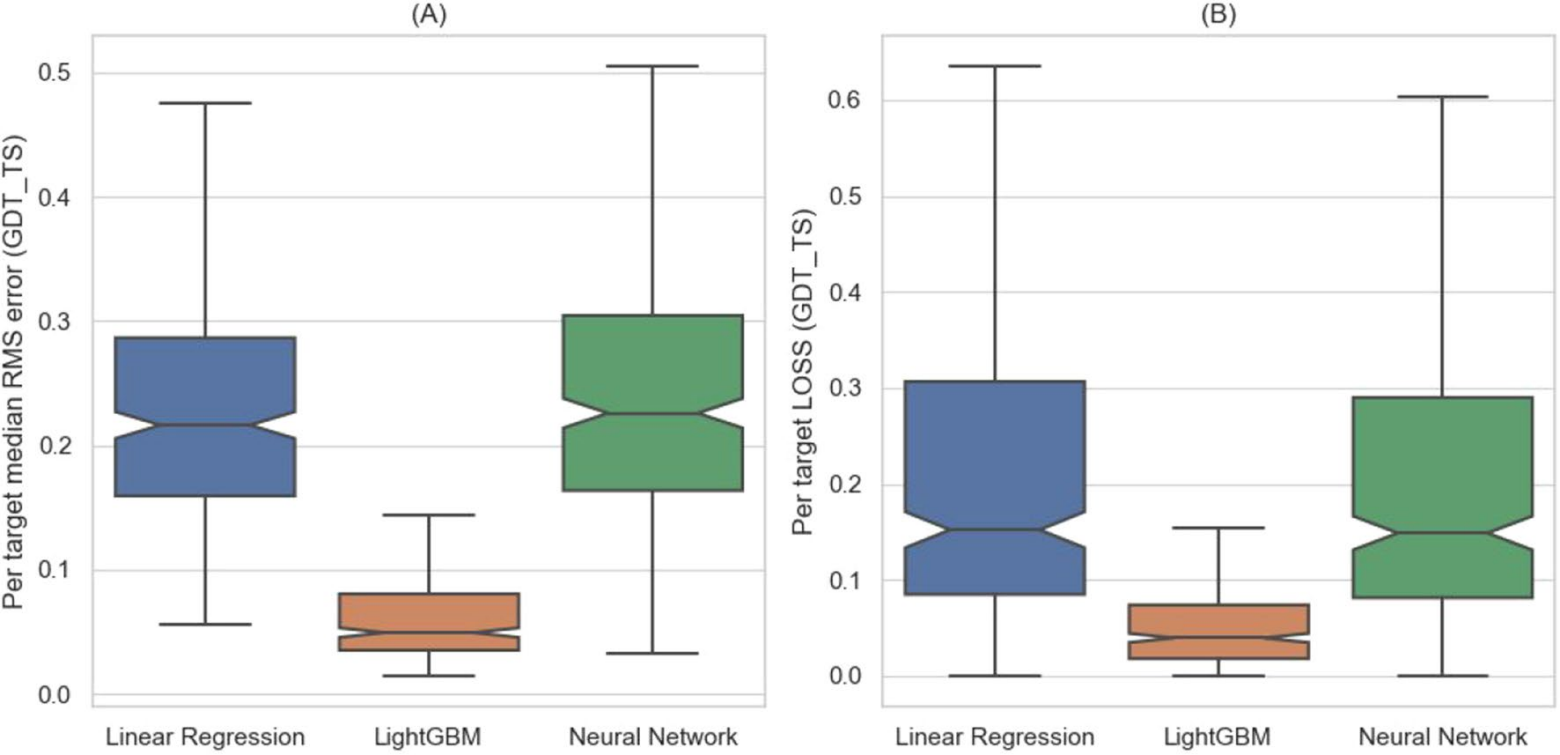
LightBGM^61^ outperforms linear regression and neural networks. The box plots depict the results of Leave-One-Target-Out experiments in terms of RMSE **(A)** and LOSS **(B)**, using three different regressors. The difference in performance between LightBGM and the two other methods is statistically significant (Wilcoxon one-sided test with a p-value < 1.39e−51). The performances of few other tree-based methods were practically indistinguishable from LightBGM (data not shown) but computation time was much longer.

In this work, we formulate EMA as a regression problem that maps measurable features of the structural models to a continuous quality score (GDT_TS) between the models and the native structures. To this end, we tested three regressors: linear regression, Light Gradient Boosted Machine (LightGBM) regressor^61^, and a fully connected neural network. The superior performance (Fig. 2) of LightGBM motivated us to examine five other tree-based regressors: BaggingRegressor, GradientBoostingRegressor, RandomForestRegressor, and ExtraTreesRegressor from the scikit-learn library^62^, as well as Extreme Gradient Boosting (XGB) regressor^63^. We remained with LightGBM, however, as it outperforms all five by a small margin and is faster to train.

#### Hyperparameters

We used grid search to find the optimal values for two hyperparameters of LightGBM regressor: learning rate and the number of estimators. Learning rate 0.1 and 100 estimators achieved good results in a reasonable computation time. For all the other parameters, we used the default values as supplied by the LightGBM framework. Specifically, the loss function that the regressor training minimizes is the RMSE.

#### Data filtering

**Figure 3:**
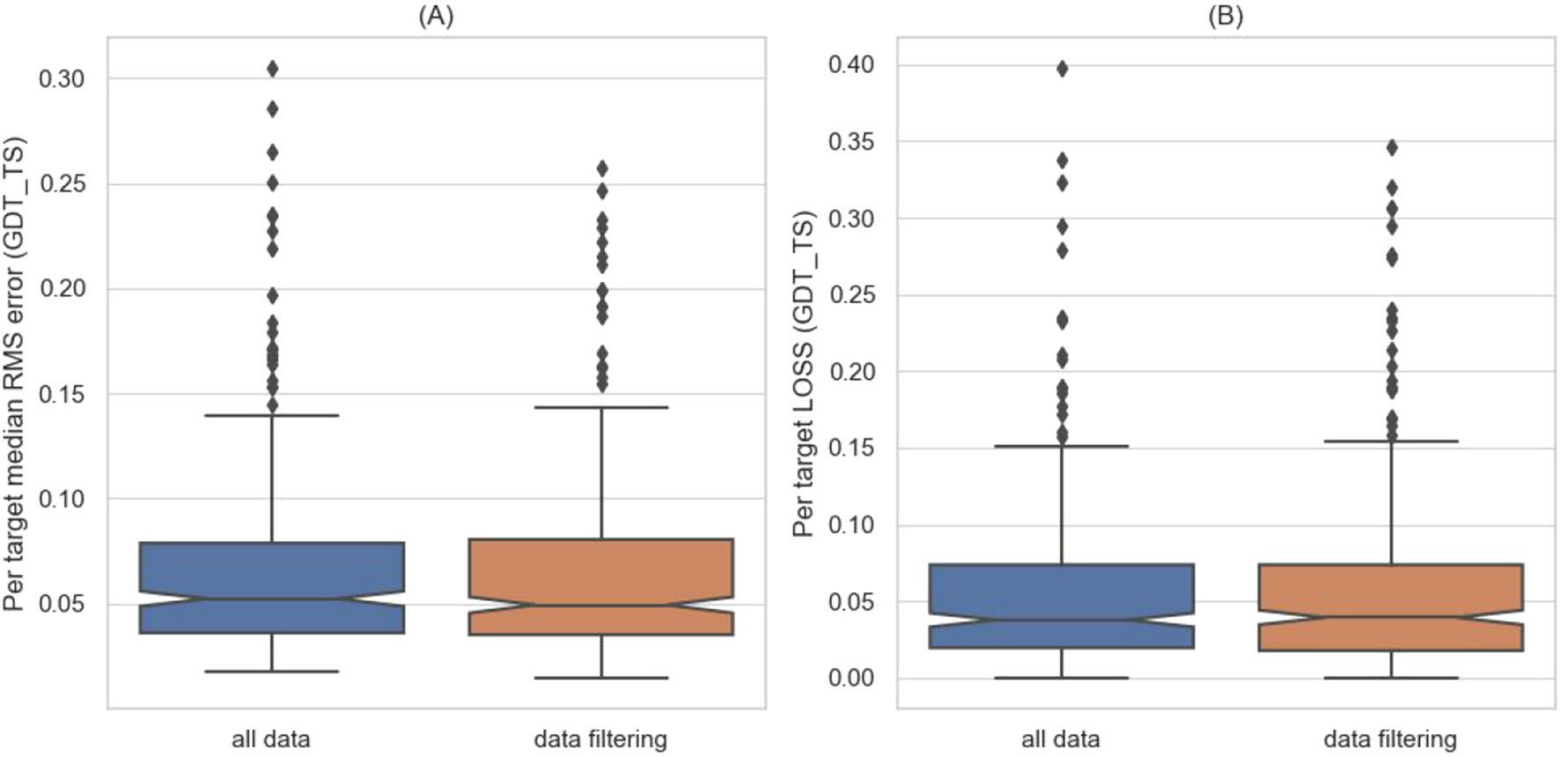
Filtering out outlier targets from the *training set* reduces the error in quality estimation (**A**), and does not affect the identification of the best models (**B**). The box plots depict the results of Leave-One-Target-Out experiments with and without data filtering in the training set. **(A)** The median of the RMSE is significantly reduced by data filtering (Wilcoxon one-sided test, with a p-value of 0.005). **(B)** Data filtering does not affect the distribution of LOSS (the quality differences between the top-ranking model, and the best model in the set). Many of the worse performing outliers in the plots are proteins that were filtered out from the training set.

Some of the dataset targets are isolated chains of multi-subunit complexes (e.g., single helices of helix-bundles). Estimating their quality is a challenge due to hydrophobic interface residues that are superficially exposed when seen out of context. For such targets, MESHI may produce feature values that are inconsistent with the label (GDT_TS), increasing noise and impairing the learning process. The qualities of such targets are hard to estimate, even in an over-fitting scenario. The removal of 18 such targets (Table S1) *from the training set* significantly reduces the median error of quality estimates and does not affect the identification of the best models (Fig. 3).

## Results

MESHI_consensus was developed using a dataset of CASP server models that were generated as blind predictions in five consecutive CASP experiments (9–13). CASP14 models serve as the ultimate test set as their true qualities were unknown at the time of prediction. Here we present the method’s performance in predicting the accuracies of models in the dataset, consider the contributions of different feature types, and conclude by presenting, and discussing CASP14 performance.

### Dataset performance

**Figure 4:**
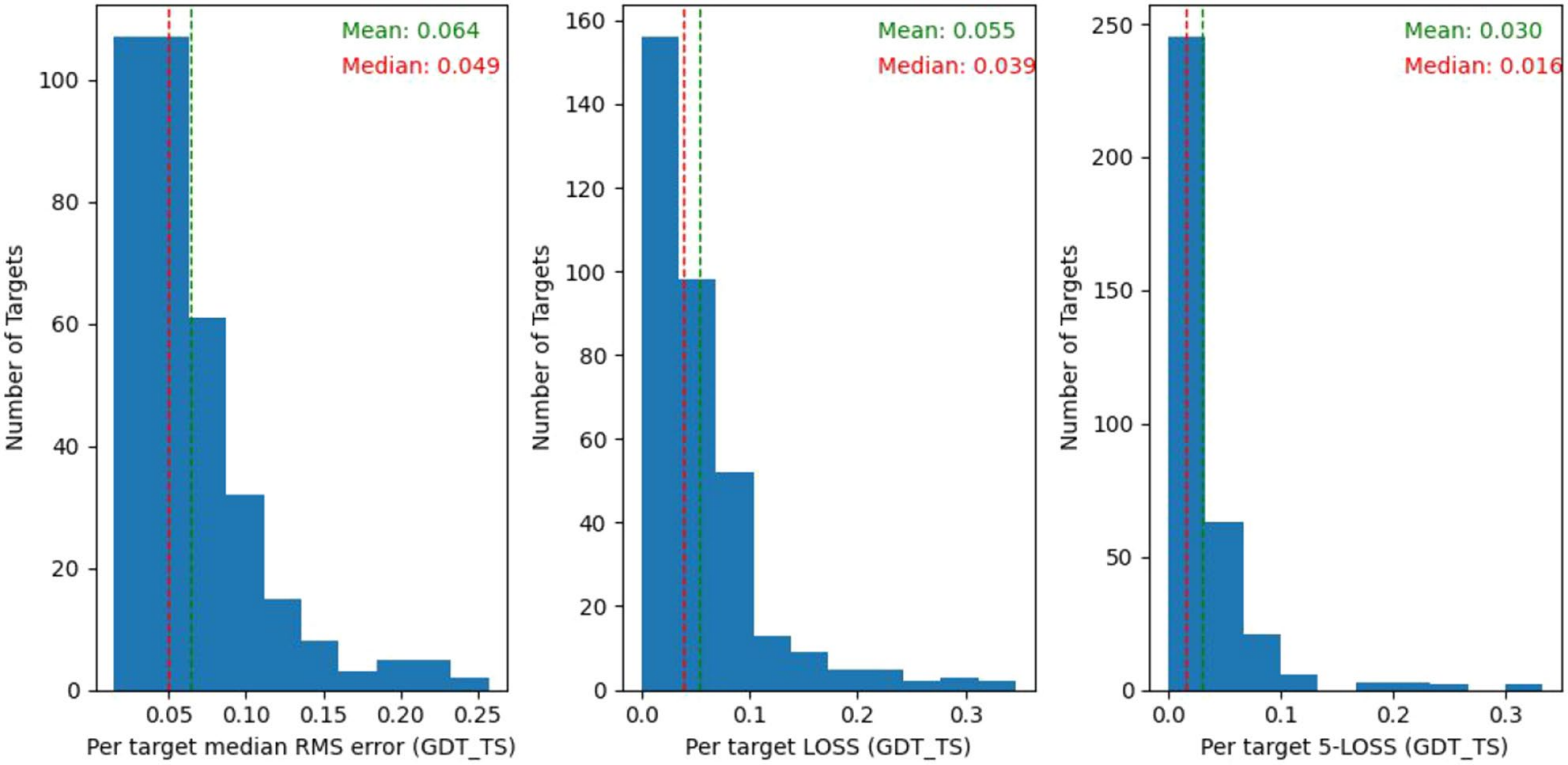
Benchmark performance of MESHI_consensus. The results of 345 leave-one-target-out experiments are summarized in three histograms: **(A)** RMSE; **(B)** LOSS, the difference between the quality of the top-ranking model and the quality of the best one. **(C)** LOSS5, the minimal difference between the qualities of 5 top-ranking models and the quality of the best one. Median (red) and mean (green) values are indicated by vertical lines.

The performance of MESHI_consensus is estimated by Leave-One-Target-Out experiments on the structural models dataset. The qualities of about half of the targets are estimated well (Fig. 4), with small (< 0.05) RMSE, and low (<0.04) LOSS. A small fraction of the targets (< 6%) are practically missed, with RMSE or LOSS above 0.2.

### Feature importance

**Figure 5:**
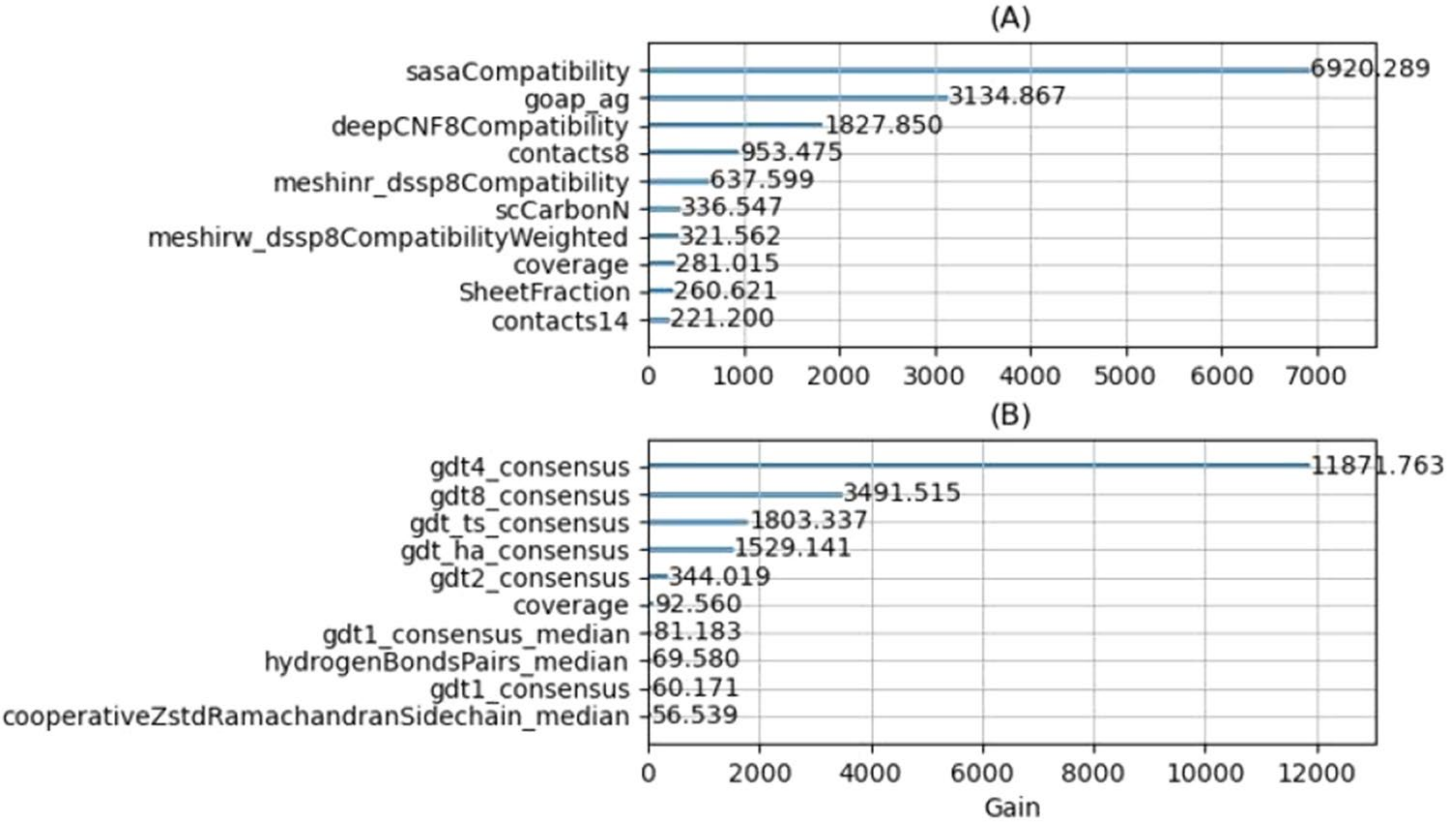
The ten most important (highest “GAIN”) features considering only the basic features **(A)** and all features **(B)**. The features from top to bottom: sasaCompatibility—a measure of the agreement between the solvent accessible surface area of the model’s residues (as measured by DSSP^65^) and their predicted accessibility^56^. goap_ag—a pairwise orientation-dependent knowledge-based potential^40^. deepCNF8Compatibility—a measure of the agreement between the secondary structure (8 states) of the model’s residues (as measured by DSSP^65^) and their predicted secondary structure^66^. contacts8 and contacts14—the average numbers of contacts with thresholds of 8 Å and 14 Å, respectively, between carbon atoms. meshinr_dssp8Compatibility and meshirw_dssp8Compatibility_Weighted—two slightly different measures of the agreement between the secondary structure (8 states) of the model’s residues (as measured by DSSP^65^) and their predicted secondary structure^59^. scCarbonN—the number of carbon atoms in the model’s side-chains. coverage—the fraction of the target sequence, which the are modeled. SheetFraction—the fraction of beta-sheet resides within the residues with any secondary structure. consensus features—see Eqs. (1–4). gdt1_consensus_median—the median value of gdt1_consensus, among all the models of a specific target. hydrogenBondsPairs_median—the median value of a cooperative hydrogen bonds energy term^67^ among all the models of a specific target. cooperativeZstdRamachandranSidechain_median—the median value of a cooperative torsion angle energy term, among all the models of a specific target.

**Figure 6:**
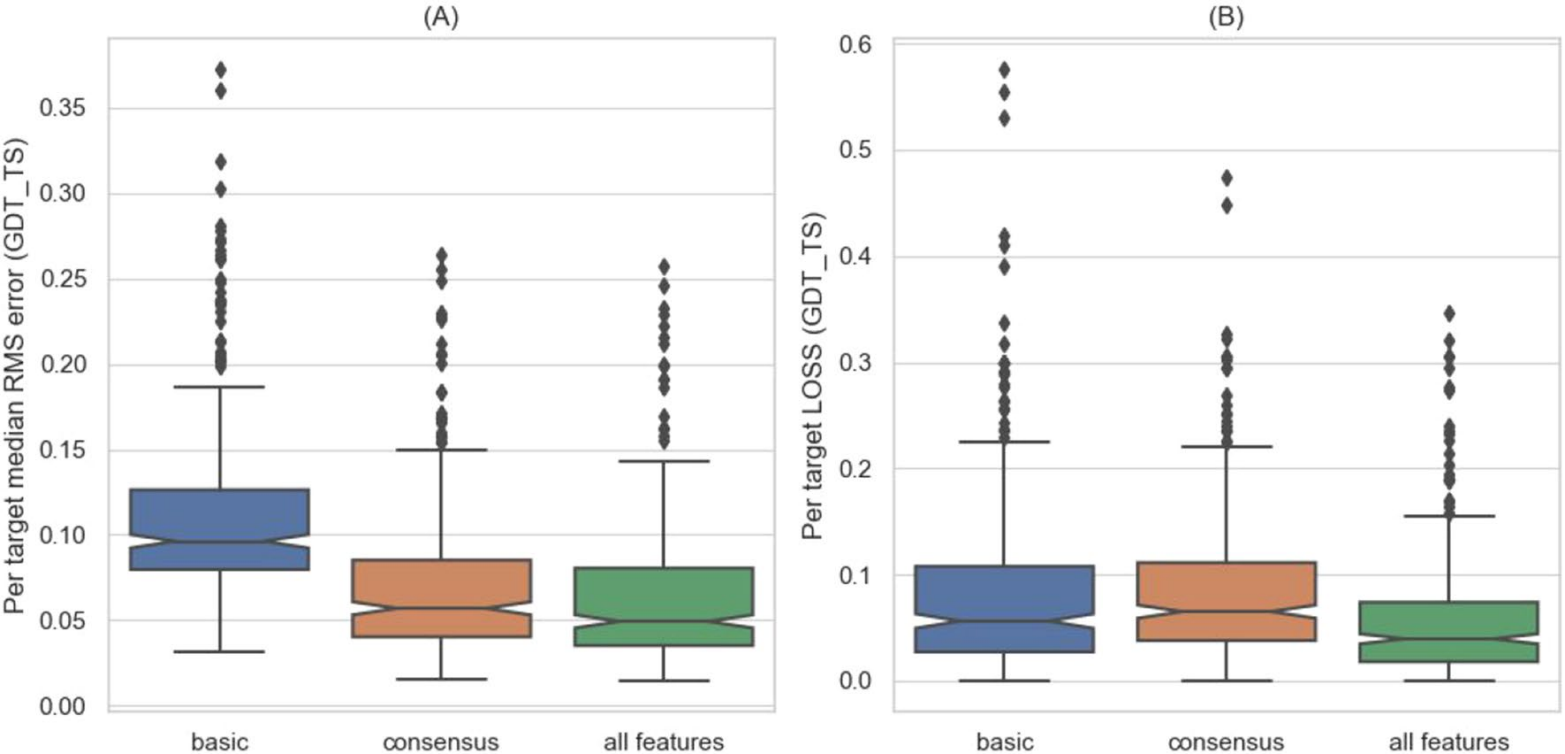
Per-target performance with different feature sets. The box plots show the per-target performance in terms of RMSE **(A)** and LOSS **(B)**, of Leave-One-Target-Out experiments with the basic features (blue), consensus features (orange), and all features (green). The differences between the median performances of all the features and the median performances of the feature subsets are statistically significant (Wilcoxon one-sided test with a p-value <10e−4).

MESHI_consensus uses a large number of features for the estimation of model accuracies. To assess their contributions to the LBGM statistical model, we first checked the importance ranking of the basic features and all the features. Following the results (Fig. 5), we also performed Leave-One-Target-Out experiments with only the basic features, only the consensus features, and with all the features (Fig. 6). Additional subgroups of features were tested (data not shown), but the best result was obtained when we used the whole set of features. The measure we used for the importance of the features is ”GAIN”, which is the sum of the information gains of all splitting points that use that feature, where information gain is the Kullback–Leibler^64^ divergence of the data before and after the split. A higher value of this metric, when compared to another feature, implies it is more important for the predictive model.

The ten highest importance features in LightGBM models that use either all the features or only the basic ones are depicted in Fig. 5. These models were trained on all the benchmark targets. Qualitatively similar estimates of feature GAINs, derived from benchmark subsets are reported in Feature importance file (supplementary material). All but one of the LightGBM models make use of the possibility to distinguish between structural models from different targets. When target-specific features are available to the models, they choose from a wide variety, without a clear preference. The basic features were not intended to include such features explicitly, yet the models assign relatively high importance to the total number of side-chain atoms in the model. This feature seems almost arbitrary and was added to the basic features by mistake (being a component of other features). We speculate that the models consistently chose it as it allows target distinction. When all features are considered, consensus features are ranked highest by a large margin, apparently, because good (i.e, close to native) models are similar to one another (they are all similar to the native structure), while low-quality models can be very different from each other. This observation is consistent with the dominance of consensus-based methods in the EMA field. When only the basic features are considered, two classes of features become dominant. The first class (e.g., sasaCompatibility), quantifies the agreement between the observed and predicted solvent exposed area and the secondary structure of model residues. Apparently, an inability to reproduce them is a strong indicator of low model quality. This is consistent with the disruptive effect of out-of-context targets (e.g., isolated subunits) on learning.

The second important class of basic features (e.g., goap_ag) rewards structural traits that are common in native structures. One common trait is compactness which manifests itself by a large number of atom contacts. The other common trait is the abundance of specific atomic interactions (e.g., contacting side-chain atoms of hydrophobic residues). Having many favorable interactions, and a compact structure are strong indicators of high model quality. Finally, as our quality measure, GDT_TS between the models and the native structures represents the fraction of accurately modeled residues, it is bound by the fraction of the target sequence which is actually modeled. Coverage features depict this fraction and are consistently ranked among the ten most important.

Considering the dominance of consensus features, we tested whether the other features are needed at all. To this end, we performed a Leave-One-Target-Out experiment with three different sets of features (Fig. 6). The first experiment served as a baseline and included only the basic features. The second experiment used only the consensus features and the third used all the features. In both, the RMSE and LOSS, the best results are obtained by using the entire set of features. For RMSE (A), which is the loss function of the regressor, most of the improvement is due to the consensus features, consistent with the ”GAIN” results (Fig. 5). Yet by using all the features we obtain statistically better performance. For LOSS (B), the basic features outperform the consensus ones, reflecting the difficulty of consensus features to identify exceptionally good models. The best structural model of target T0581 for example (BAKER-ROSETTASERVER_TS4, 0.64 GDT_TS) was picked by the statistical model that was trained with the basic features only. Training with all the features resulted picking the second-best model (GDT_TS 0.33), which is similar to some other inaccurate ones. Notably, however, adding consensus features to the basic ones reduces the number of outliers and the magnitude of their deviation from the median performance. This is probably because consensus features are indifferent to complex subunits and membrane proteins that distort many basic features.

### CASP14: MESHI_consensus

The previous round of CASP experiments, CASP14 (May–August, 2020) serves as the independent test set of this study. MESHI_consensus took part in that experiment as an EMA server and submitted 11,135 global quality predictions of server models.

**Figure 7:**
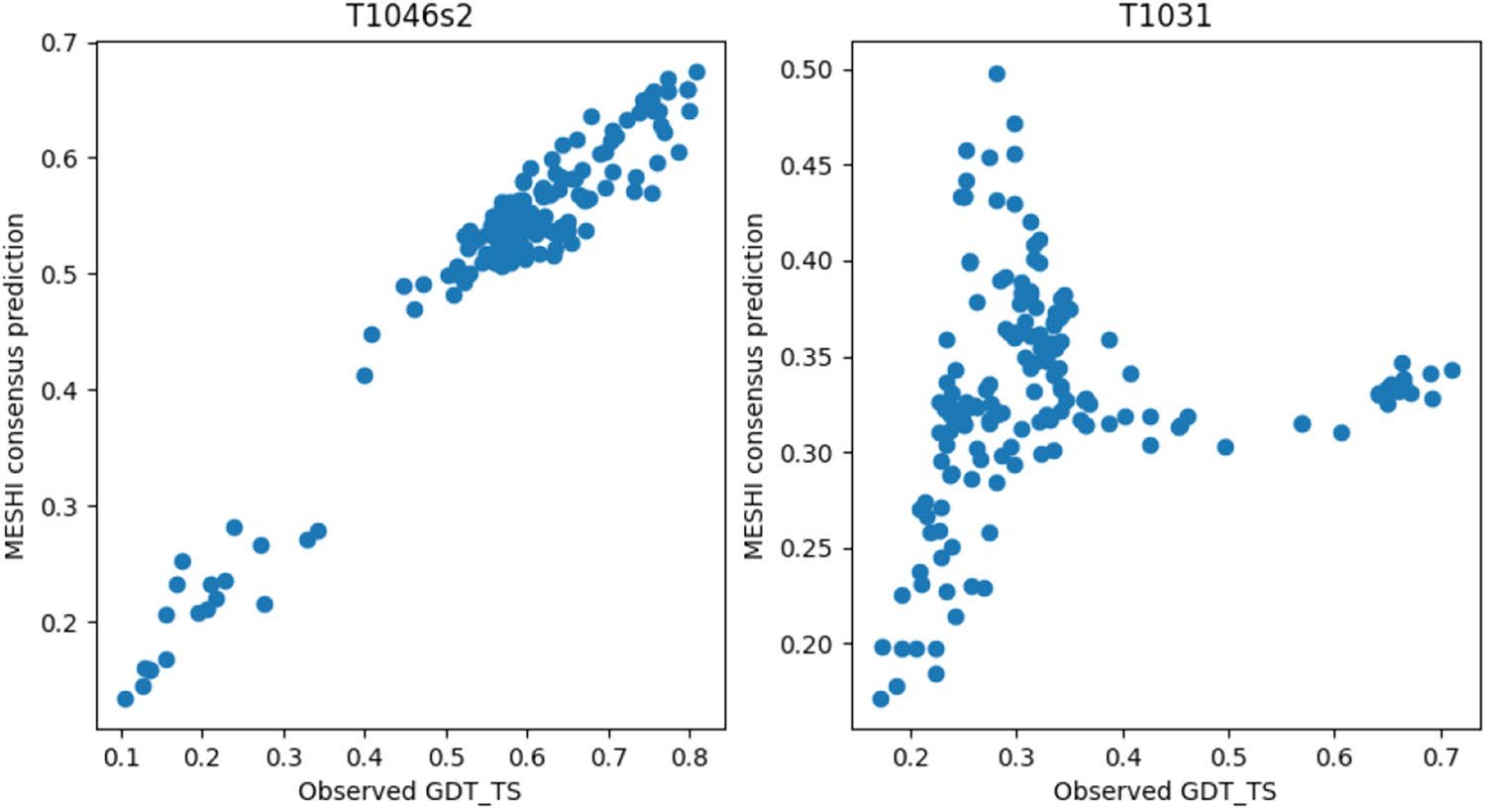
Examples of MESHI_consensus success and failure in CASP14. Predicted vs. observed qualities of models from two targets: Left: For target T1046s2 (PDB code: 6px4), MESHI_consensus reached a low RMSE (0.074) and the top-scoring model is indeed the best one (zero LOSS). Right: For target T1031 (PDB code: 6vr4) the RMSE is 0.128 and the best model ranked very low (LOSS is 0.428)

**Figure 8:**
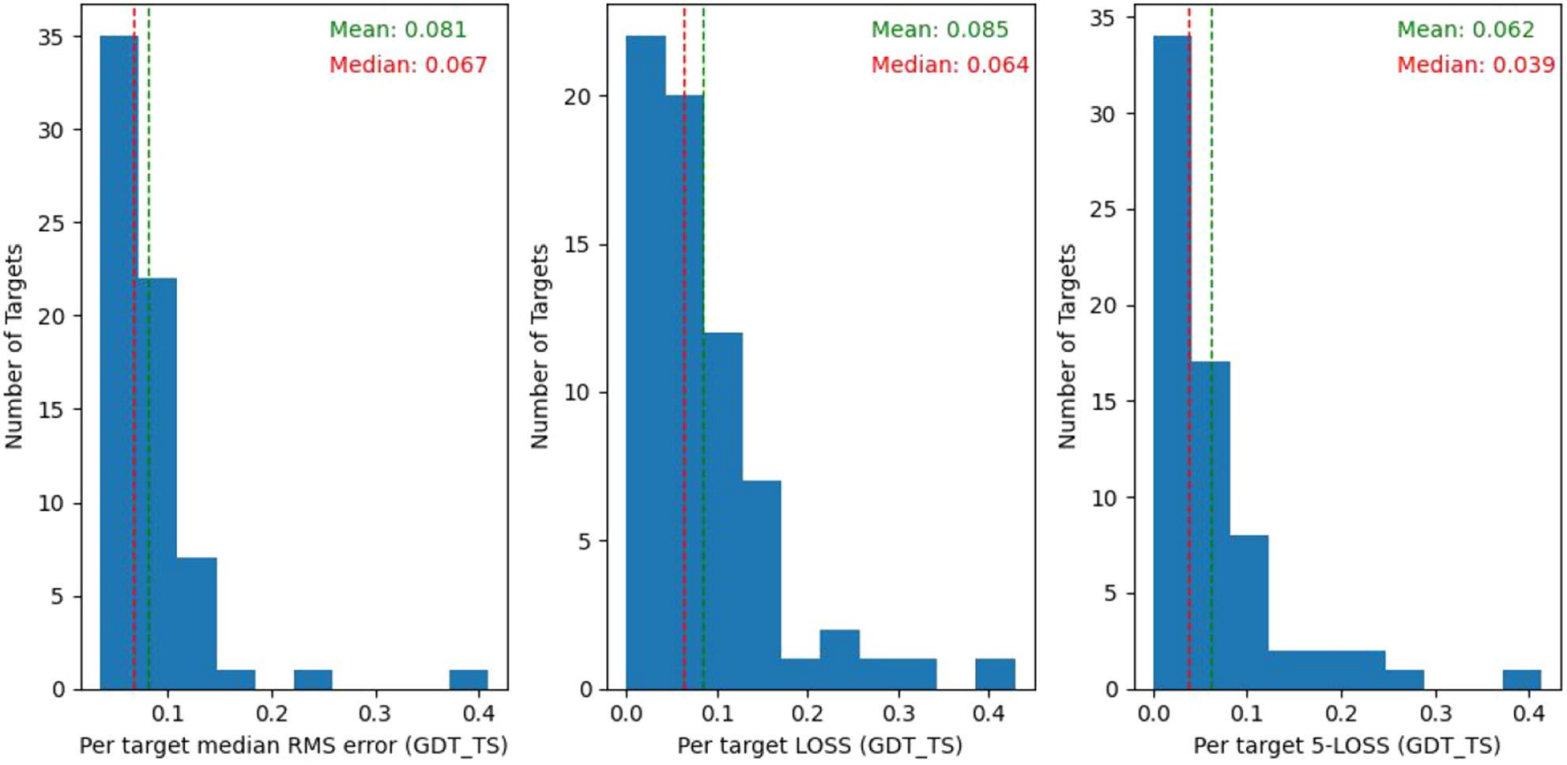
The model accuracies of most (>50%) of the CASP14 targets can be predicted within an error of 0.07 GDT_TS units. The plots depict the results of MESHI_consensus method in CASP14. Median (red) and mean (green) values are indicated by vertical lines.

One target, T1093, was missed due to technical failure. Figure 7 depicts the best (left) and worst (right) results with targets T1046s2 and T1031 respectively. The overall performance on this test set (Fig. 8) is comparable to that of the benchmark (Fig. 4). The slight performance reduction is discussed below. Comparison with the other 71 research groups that competed in the EMA category reveals a state-of-the-art performance, with more than 65% of the predictions ranked within the top 10 in either LOSS or RMSE. Specifically, MESHI_consensus reached:

- Top GDT_TS MCC(50) score (Table 1A)
  (https://predictioncenter.org/casp14/qa_aucmcc.cgi)
- Third lowest average prediction error (Table 1B)
  (https://predictioncenter.org/casp14/qa_diff_mqas.cgi.
- Sixths lowest average LOSS (Table 1C)
  (https://predictioncenter.org/casp14/qa_diff2best.cgi).

Notably, among the other top-performing methods, one (MESHI) is a curiosity-driven variant of MESHI_consensus variant, that simply adds server names as a feature and ranked a bit higher.

**Figure 9:**
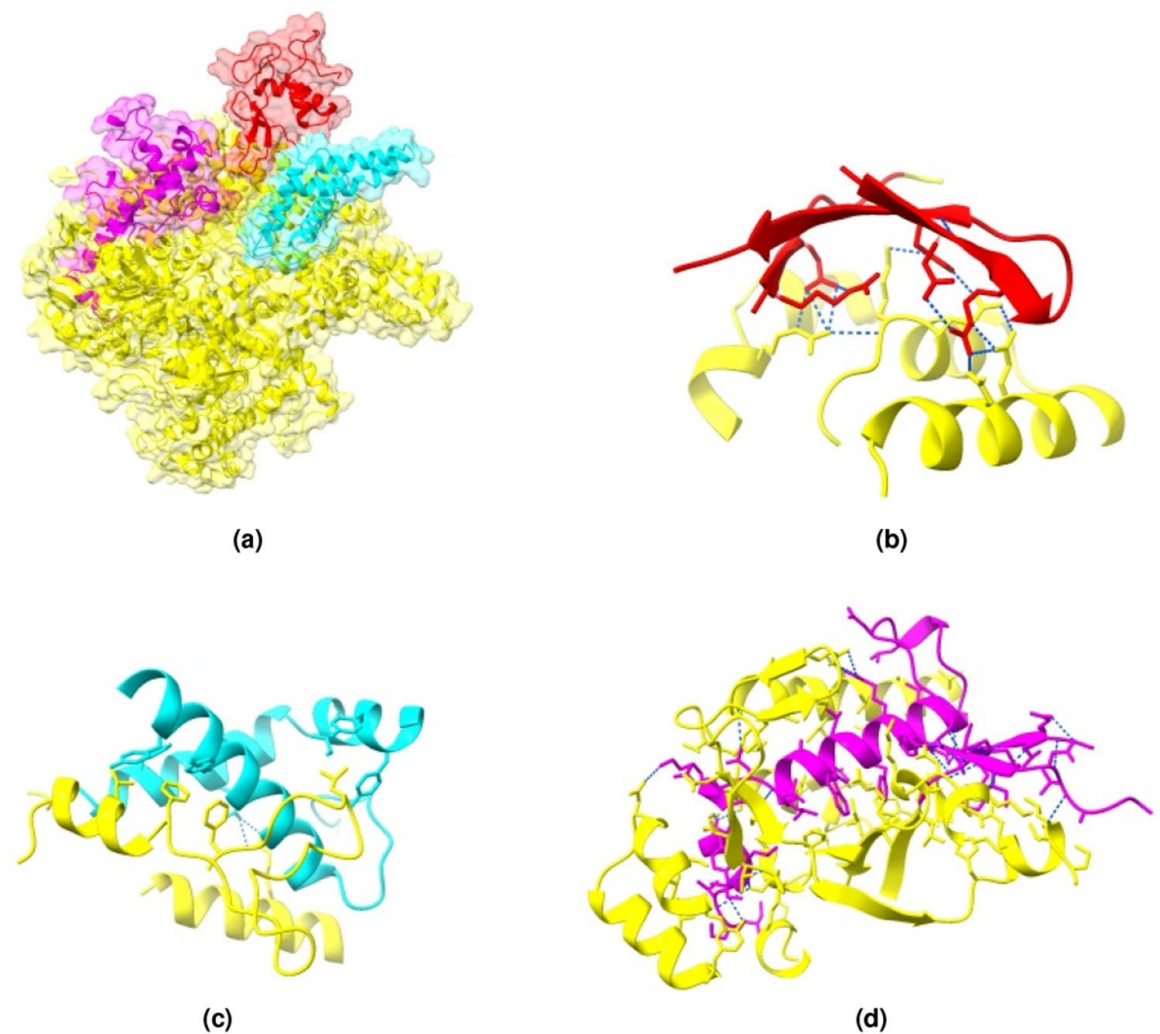
Three examples of apparent bias of EMA prediction, by the absence of molecular context. The figure presents the large (2225 residues) DNA-dependent RNA polymerase of crAss-like phage phi14:2 (PDB 6VR4) that gave rise to ten CASP14 targets: The whole protein (T1044), which was not offered as an EMA target, and nine domain targets T1031, T1033, T1035, T1037, T1039, T1040, T1041, T1042, and T1043. For six of them: T1031, T1033, T1035, T1039, T1040, and T1043, MESHI_consensus failed to provide reliable EMA predictions (LOSS above 0.16). We speculate that these failures may be attributed, at least partially, to the absence of the protein context in the isolated domains. (**a**) The three domain targets, depicted in the context of the whole protein (yellow): T1031 (residues 1–95, red), T1033 (residues 96–196, cyan), T1040 (residues 1372–1501, magenta). (**b–d**) The three interfaces of these domains (respectively) with the rest of the protein. A sample of the interacting residues is shown, the rest are hidden for clarity. Blue dashed lines represent hydrogen bonds and salt bridges (Figure is drawn with ChimeraX^68^).

Notwithstanding these achievements, the overall performance of MESHI_consensus in CASP14 (Fig. 8) is worse than in the dataset’s Leave-One-Target-Out experiments (Fig. 4). Notably, nine of the EMA targets are domains of a single large protein (Fig. 9). MESHI_consensus failed to predict six of them with LOSS values ranging from 0.16 to 0.4 (Fig. 7, right). We speculate that the lack of the protein context had to do with the poor performance, as no other targets showed so high LOSS values. As demonstrated in Fig. 9, these structures have numerous inter-domain stabilizing interactions, that are missing in the isolated EMA targets. A similar phenomenon is also observed in the dataset (see the Data filtering section). Target T1073 also showed exceptionally bad performance with an RMSE of 0.41. Unfortunately, we cannot study this case, as its structure has not been published yet. Another, more speculative explanation for the lower performance is the methodological turning point of CASP14 (discussed below). It raises a major challenge to MESHI_consensus, as well as to any supervised learning method that uses sets of CASP server models training. The test (CASP14 models) and training sets were not sampled from the same distribution, as the models of CASP14 are on average more accurate than those of previous CASP experiments.

**Table 1:** CASP14 performances.

A	
Group name	MCC(50)
MESHI_consensus	0.746
MESHI	0.742
DAVIS-EMAconsensus	0.728
ModFOLDclust2	0.724
MUfoldQA_G	0.723
EMAP_CHAE	0.707
UOSHAN	0.696
Yang_TBM	0.692

B	
Group name	RMSE
DAVIS-EMAconsensus	0.0673
MUfoldQA_G	0.0723
MESHI_consensus	0.0724
MESHI	0.0725
ModFOLDclust2	0.0735
EMAP_CHAE	0.0739
Yang_TBM	0.0804
UOSHAN	0.0836

C	
Group name	LOSS
MULTICOM-CONSTRUCT	0.0735
MULTICOM-AI	0.0792
MESHI	0.0793
MULTICOM-CLUSTER	0.0802
MUfoldQA_G	0.082
MESHI_consensus	0.084
BAKER-ROSETTASERVER	0.084
BAKER-experimental	0.0845

The tables present the top-scoring groups^31,71–75^ by three measures: MCC(50) (A), RMSE (B), and LOSS (C). Results are reproduced from the CASP website at (https://predictioncenter.org/casp14). Note that in the CASP site RMSE and LOSS are referred to as ”differences (predicted vs observed)” and ”difference from the best”, respectively, and their performances are depicted as percentages. The servers ”Seder2020” and ”Seder2020hard” that submitted an EMA prediction for a single target were omitted.

**Figure 10:**
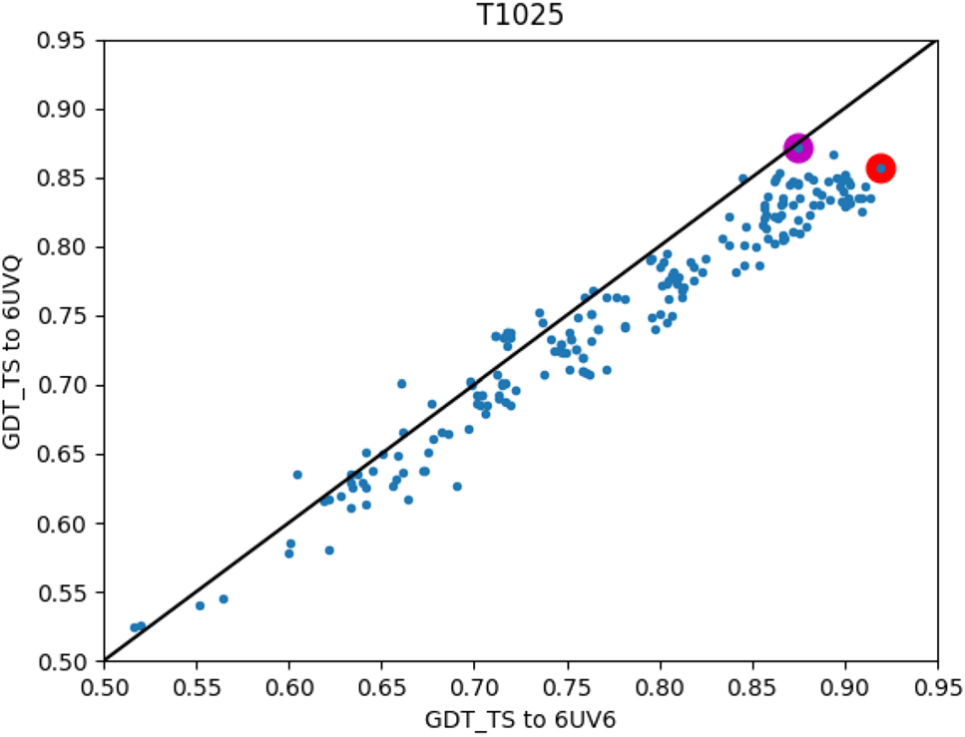
Model accuracy with respect to two alternative native structures. Target T1025 was evaluated by the CASP14 assessors with the ligand-bound D-glucose *O*-methyltransferase as its native structure (PDB entry 6UV6^69^). The structure of the unbound protein is also available (PDB entry 6UVQ^70^). While the choice of either structure is arbitrary, the resulted performance measures are quantitatively different. A theoretical EMA oracle that provides 6UVQ based accuracy as its prediction, would have 4% RMSE and 6% LOSS. The dots represent server models, the black line is the diagonal (x $=$ y), and the red and magenta circles depict the top model by 6UV6 and 6UVQ respectively.

## Discussion

This manuscript introduces MESHI_consensus, a new method for quality assessment of protein models. MESHI_consensus uses a large and diverse set of features, representing both physical concepts (e.g., energy terms) and domain knowledge (target-specific and consensus-based features). The features are integrated to a single score by a powerful and computationally efficient, tree-based LightGBM regressor^61^. One type of state-of-the-art features, which the method lacks, is compatibility with predicted distances derived from multiple sequence alignments (*MSA*)^11,76^. MSA-driven distances are the keystone of the current breakthrough in PSP, and compatibility with them seems to be a powerful feature^20^.

The development of MESHI_consensus was guided by Leave-One-Target-Out experiments using a non-redundant dataset of structural models from previous CASP experiments. The recent CASP14 provided an objective performance test and MESHI_consensus scores among the top methods (see Results). One may speculate that had we used contact compatibility features we could perform better. During the study, we invested much effort in analyzing failures, that is targets for which we considerably missed the actual model qualities and/or their rankings. Many of these failures could be rationalized in retrospect as related to the inability of our features to consider stabilizing inter-molecular interactions. We tried to implement insights from this analysis through data filtering with limited success. One may hope though that a more systematic approach to this problem may lead to better performance in future studies.

A profound limitation of MESHI_consensus, is the reliance on a single native structure as the gold standard for the labeling of the data. This is in line with the common practice in the field, which is applied in CASP and in all the studies that we are aware of. Yet, this practice ignores the structural flexibility of proteins as manifested by diverse structures of the same protein in different PDB entries^77^, and in the results of NMR studies. Figure 10 demonstrates this observation by assessing the server models of target T1025 using the ligand-bound and apo crystal structures of that protein. Target T1064 shows a similar, yet less pronounced trend (data not shown). In the CASP context alternative structures are rarely available, and thus ignoring them is practically unavoidable, and can be seen as part of the ”noise” characteristic of any experiment. In the training phase, however, ignoring available knowledge of structural multiplicity adds superficial, arbitrary, constraints to the learning process, and probably harms the resulted statistical model. Structural multiplicity can be introduced into the training phase of EMA methods if structural models are evaluated by their similarity to the closest of the known structures, rather than to a single arbitrary one. We have already demonstrated the usefulness of a similar approach in the related fields of secondary structure prediction^78^ and knowledge-based energy functions^79^.

Finally, CASP14 has witnessed a remarkable breakthrough in PSP, with many models of hard targets reaching experimental quality. This achievement had a limited effect on the EMA section of CASP14 as the cutting-edge method, AlphaFold^16^, did not provide a server. Yet, it is evident that a new standard of model qualities is set^80^. Will EMA be needed at all when the models are ”almost perfect”? An obvious answer is that we are in the middle of the event and its consequences cannot be predicted. More fundamentally, the new achievements seem to open new horizons for PSP, considerably improving our ability to cope with essential problems like structures of molecular complexes and protein dynamics. These challenges require their own EMA tools.

We believe that the insights of this study, most importantly the central role of structural multiplicity and molecular context, will gain much importance in the era of high-accuracy modeling. On a more speculative note, we suggest that features like the ones used in this and related studies will also remain relevant, as design principles for new, probably neural-network-based, architectures. The current application of neural network techniques to EMA use standard architectures and avoid ”feature engineering”, such as energy terms. One may speculate that introducing more domain knowledge into the network architecture and its input features will result in more accurate and stable performance.

## Supplementary Information

Supplementary Information.

Supplementary Table S1.

## Data Availability

The data sets generated and analysed during the current study are available at http://meshi1.cs.bgu.ac.il/BittonAndKeasar2021/.
