# Supplementary figures and images for: Estimation of model accuracy by a unique set of features and tree-based regressor
Source: Sci Rep. 2022 Aug 18;12:14074. doi: 10.1038/s41598-022-17097-z (PMC9388490; doi:10.1038/s41598-022-17097-z)

## Feature importance file

1.

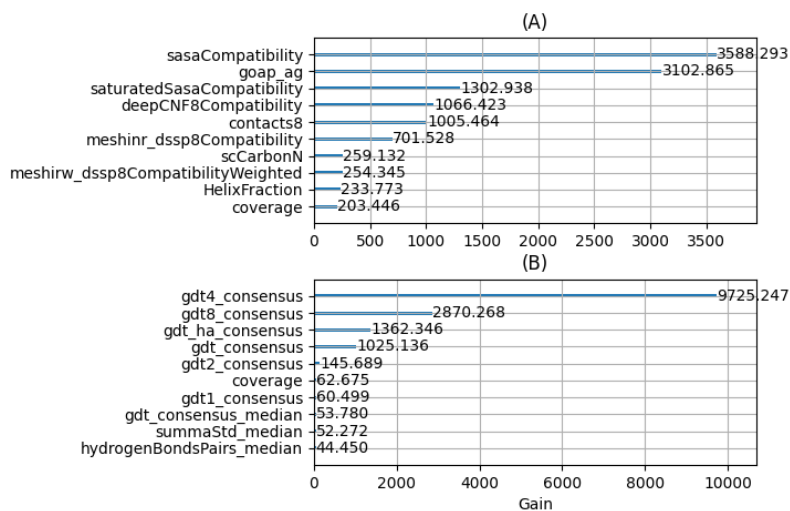

2.

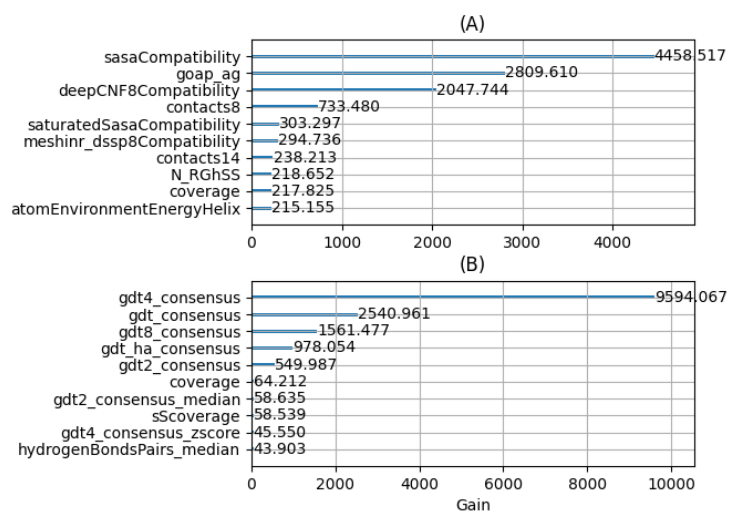

3.

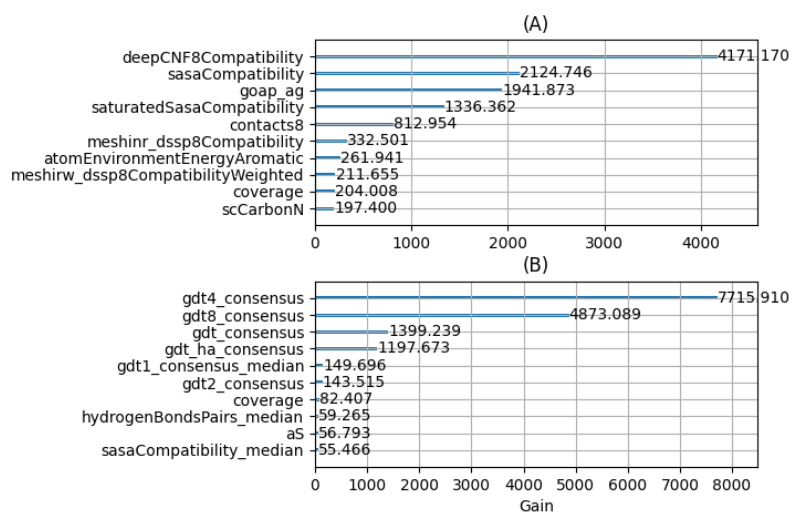

4.

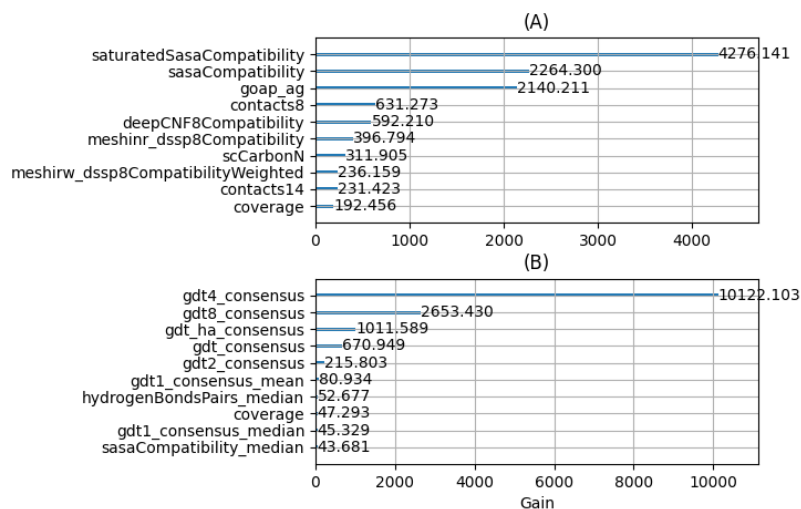

5.

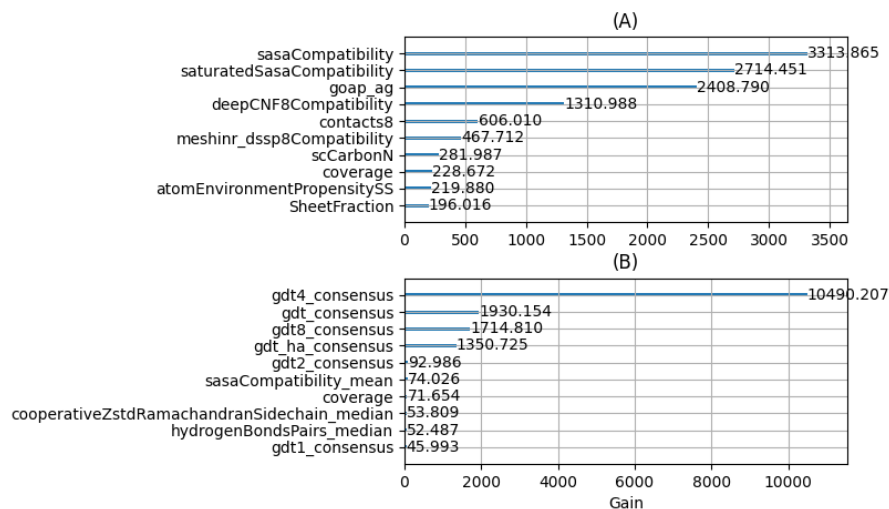

Supplement: Supplementary file 1 — Supplementary Information. [file 41598_2022_17097_MOESM1_ESM.pdf]
