# Supplementary material for: Estimation of model accuracy by a unique set of features and tree-based regressor
Source: Sci Rep. 2022 Aug 18;12:14074. doi: 10.1038/s41598-022-17097-z (PMC9388490; doi:10.1038/s41598-022-17097-z)
Supplement: Supplementary file 2 — Supplementary Table S1. [file 41598_2022_17097_MOESM2_ESM.pdf]

Table S1 – Targets list

Target name	CASP competition	Target name	CASP competition	Target name	CASP competition
T0517	9	T0561	9	T0597	9
T0520	9	T0562	9	T0598	9
T0521	9	T0564	9	T0599	9
T0522	9	T0566	9	T0600*	9
T0523	9	T0567	9	T0601	9
T0525	9	T0568	9	T0602	9
T0528	9	T0569	9	T0603	9
T0529	9	T0570	9	T0604	9
T0530	9	T0571	9	T0605*	9
T0531	9	T0572	9	T0606	9
T0533	9	T0573	9	T0607	9
T0534	9	T0574	9	T0608	9
T0535*	9	T0575	9	T0609	9
T0536	9	T0576	9	T0610	9
T0537	9	T0577	9	T0611	9
T0538	9	T0578	9	T0612	9
T0539	9	T0579	9	T0614	9
T0540	9	T0580	9	T0615	9
T0541	9	T0581	9	T0616	9
T0542	9	T0582	9	T0617	9
T0545	9	T0584	9	T0618	9
T0546*	9	T0585	9	T0619	9
T0547	9	T0586	9	T0620	9
T0548	9	T0587	9	T0621	9
T0549	9	T0588	9	T0622	9
T0550	9	T0589	9	T0623	9
T0551	9	T0590	9	T0624	9
T0555	9	T0592	9	T0625	9
T0557	9	T0593	9	T0626	9
T0558	9	T0594	9	T0627	9
T0560	9	T0596	9	T0628	9

* - Targets that are exclude from the training set.

Target name	CASP competition	Target name	CASP competition	Target name	CASP competition
T0629	9	T0676	10	T0720	10
T0630	9	T0678	10	T0721	10
T0632	9	T0680	10	T0722	10
T0634	9	T0681	10	T0723	10
T0635	9	T0682	10	T0724	10
T0637	9	T0683	10	T0726	10
T0638	9	T0684	10	T0727	10
T0639	9	T0685	10	T0729	10
T0641	9	T0687	10	T0731*	10
T0643	9	T0688	10	T0732	10
T0644	10	T0689	10	T0733	10
T0648	10	T0691	10	T0734	10
T0649	10	T0692	10	T0735	10
T0651	10	T0693	10	T0736	10
T0652	10	T0694	10	T0737	10
T0654	10	T0695	10	T0742	10
T0655	10	T0696*	10	T0743	10
T0657	10	T0697	10	T0744	10
T0658	10	T0698	10	T0745	10
T0659*	10	T0700	10	T0746	10
T0661	10	T0703	10	T0747	10
T0662*	10	T0704	10	T0749	10
T0663	10	T0705	10	T0750	10
T0665	10	T0706*	10	T0751	10
T0666	10	T0708	10	T0752	10
T0667	10	T0710	10	T0753	10
T0668	10	T0711	10	T0754	10
T0672	10	T0712	10	T0755	10
T0673	10	T0715	10	T0756	10
T0674	10	T0716	10	T0757	10
T0675	10	T0717	10	T0758	10

* - Targets that are exclude from the training set.

**Target name**	**CASP competition**	**Target name**	**CASP competition**	**Target name**	**CASP competition**
T0759*	11	T0794	11	T0841	11
T0760	11	T0796	11	T0843	11
T0761	11	T0797*	11	T0845	11
T0762	11	T0798	11	T0846	11
T0763	11	T0799	11	T0847	11
T0764	11	T0800	11	T0848	11
T0765	11	T0802	11	T0849	11
T0766	11	T0803	11	T0851	11
T0767	11	T0806	11	T0852	11
T0768	11	T0807	11	T0853	11
T0770	11	T0808	11	T0854	11
T0771	11	T0812	11	T0855	11
T0772	11	T0813	11	T0856	11
T0773	11	T0814	11	T0857	11
T0774	11	T0815	11	T0858	11
T0775	11	T0816	11	T0860	12
T0776	11	T0817	11	T0861	12
T0777	11	T0818	11	T0862*	12
T0780	11	T0819	11	T0865	12
T0781	11	T0821	11	T0866*	12
T0782	11	T0822	11	T0868	12
T0783	11	T0826	11	T0869	12
T0784	11	T0828	11	T0870	12
T0785	11	T0829	11	T0872	12
T0786	11	T0830	11	T0877	12
T0787*	11	T0831	11	T0878	12
T0788	11	T0832	11	T0879	12
T0790	11	T0833	11	T0882	12
T0791	11	T0834	11	T0884	12
T0792	11	T0837	11	T0885	12
T0793	11	T0840	11	T0889	12

* - Targets that are exclude from the training set.

Target name	CASP competition
T0891	12
T0893	12
T0894	12
T0895	12
T0900	12
T0902	12
T0903	12
T0904	12
T0909	12
T0912	12
T0918	12
T0920	12
T0921	12
T0922*	12
T0928	12
T0929	12
T0930*	12
T0932	12
T0933	12
T0942*	12
T0943	12
T0944	12
T0945	12
T0948	12
T0950	13
T0951	13
T0953s1	13
T0953s2	13
T0954	13
T0955*	13
T0957s1	13
T0957s2	13
T0958	13
T0960	13
T0961	13
T0963	13
T0965	13
T0966	13
T0967	13
T0968s1	13
T0968s2	13
T0969	13
T0970	13
T0971	13
T0976	13
T0980s1	13
T0980s2	13
T0984	13
T0986s1	13
T0986s2	13
T0990	13
T1000	13
T1003	13
T1005	13
T1006	13
T1008	13
T1009	13
T1011	13
T1014	13
T1016	13
T1018	13
T1021s1	13
T1021s2	13
T1021s3	13
T1022s1	13
T1022s2	13

* - Targets that are exclude from the training set.
